# Observations of the Effects of Angiotensin II Receptor Blocker on Angiotensin II-Induced Morphological and Mechanical Changes in Renal Tubular Epithelial Cells Using Atomic Force Microscopy

**DOI:** 10.1155/2018/9208795

**Published:** 2018-05-20

**Authors:** Jin Sug Kim, Gi-Ja Lee, Tae Won Lee, Chun Gyoo Ihm, Yu Ho Lee, Yang Gyun Kim, Ju-Young Moon, Sang Ho Lee, Ji-Hye Kim, Sung-Wook Kang, Su-Jin Chae, Hun-Kuk Park, Kyung Hwan Jeong

**Affiliations:** ^1^Division of Nephrology, Department of Internal Medicine, Kyung Hee University School of Medicine, Seoul, Republic of Korea; ^2^Department of Biomedical Engineering, Kyung Hee University School of Medicine, Seoul, Republic of Korea

## Abstract

**Objective:**

Angiotensin II (Ang II) plays a profibrotic role in the kidneys. Although many pathways of Ang II have been discovered, the morphological and mechanical aspects have not been well investigated. We observed the changes in tubular epithelial cells (TECs) after Ang II treatment with or without Ang II receptor blockers (ARBs) using atomic force microscopy (AFM).

**Methods:**

TECs were stimulated with Ang II with or without telmisartan, PD123319, and blebbistatin. AFM was performed to measure the cellular stiffness, cell volume, and cell surface roughness. Epithelial to mesenchymal transition markers were determined via immunocytochemistry.

**Results:**

After Ang II stimulation, cells transformed to a flattened and elongated mesenchymal morphology. Cell surface roughness and volume significantly increased in Ang II treated TECs. Ang II also induced an increase in phospho-myosin light chain and F-actin and a decrease in E-cadherin. Ang II coincubation with either telmisartan or blebbistatin attenuated these Ang II-induced changes.

**Conclusion:**

We report, for the first time, the use of AFM in directly observing the changes in TECs after Ang II treatment with or without ARBs. Simultaneously, we successfully measured the selective effect of PD123319 or blebbistatin. AFM could be a noninvasive evaluating strategy for cellular processes in TECs.

## 1. Introduction

Renal fibrosis, characterized by increased extracellular matrix (ECM) accumulation on the kidney parenchyma, is the final common manifestation of chronic kidney disease (CKD), regardless of the primary causes [1, 2]. Previous studies reported that renal tubular epithelial cells (TECs) played an important role in the development of renal tubulointerstitial fibrosis [3]. TECs release chemokines and profibrogenic cytokines and undergo epithelial to mesenchymal transition (EMT) in pathological conditions [4–6]. Therefore, understanding the changes of TECs are important for the prevention and effective treatment of renal fibrosis.

Angiotensin II (Ang II), a major component of the renin angiotensin aldosterone system (RAAS), is known to be a crucial mediator of renal fibrosis [7, 8]. Several studies have demonstrated the ability of Ang II to induce EMT of TECs by regulating the synthesis of ECM and production of profibrotic molecules such as transforming growth factor-*β* [9]. Ang II binds to two specific receptors, angiotensin type 1 (AT_1_) and angiotensin type 2 (AT_2_) receptor [10]. AT_1_ receptor is known to mediate most of the classical physiologic and pathologic effects of Ang II, while the role of AT_2_ receptor is not completely established [11]. Many* in vitro* and* in vivo* studies have established that RAAS blockade using AT_1_ receptor blockers has therapeutic effects on renal tubulointerstitial fibrosis. [12, 13]. However, most of these studies demonstrated this mechanism in indirect ways, including gene and protein expression, associated with renal fibrosis and RAAS. Therefore, further studies with direct measurement of the morphological and mechanical changes of TECs during Ang II stimulation and treatment with Ang II receptor blockers (ARBs) are needed.

Atomic force microscopy (AFM), invented in 1986 by Binnig et al. [14], has become a useful noninvasive imaging tool in biological and medical research [15]. AFM shows the force-distance (FD) curve by measuring the force between its probe tip and the sample surface and can be used to evaluate a sample's physical properties. Hence, the stiffness and adhesive characteristics of cell membranes can be evaluated by AFM [16]. Recently, many studies suggested that the information obtained via AFM helps in understanding the biological and physical mechanism of renal injury [17, 18]. Our group previously used AFM to monitor Ang II-induced conformational changes in mesangial cells [19], and we also successfully observed that the changes in the Ang II-stimulated mesangial cells were effectively disrupted by treatment with telmisartan, a specific AT_1_ receptor blocker [20]. However, only a few studies investigated the changes of TECs using AFM. In this study, we used AFM to observe the Ang II-induced morphological and mechanical changes in TECs. Moreover, the effects of various ARBs on Ang II treated TECs were also investigated [21].

## 2. Materials and Methods

### 2.1. Cell Culture and Treatment

A well characterized, normal rat kidney cell line (NRK-52E; Sigma-Aldrich, MO, USA) was used in this study. Cells were cultured in Dulbecco's Modified Eagle's Medium (DMEM; Gibco-Invitrogen, CA, USA) containing 4.5 g/L of glucose with 10% fetal calf serum in a humidified 5% CO_2_ incubator at 37°C and passaged twice a week. NRK-52E cells between the 28th and 30th passages were used. In preparation for AFM observation, the cells were seeded into a collagen type I-coated 60 mm cell culture dish. After the cells reached confluence, they were washed once with filtered phosphate buffered saline (PBS; pH 7.4), and new DMEM was added. Cells were then incubated for 24 hours with Ang II (Sigma-Aldrich, MO, USA) in the presence or absence of telmisartan (Sigma-Aldrich, MO, USA), an AT_1_ receptor antagonist. We also used PD123319 (Sigma-Aldrich, MO, USA), an AT_2_ receptor antagonist, as a negative control and blebbistatin (Sigma-Aldrich, MO, USA), a myosin II inhibitor, as a positive control for telmisartan at the same concentration (1 × 10^−6 ^M) for 24 hours.

### 2.2. AFM Observations

Contact mode AFM images were obtained using a NANO Station II (Surface Imaging Systems, Herzogenrath, Germany). The AFM was placed on an active vibration isolation table (TS-150; S.I.S., Herzogenrath, Germany) inside a passive vibration isolation table (Pucotech, Seoul, Korea) to eliminate external noise. Silicon cantilevers with the reflective side coated with gold were used for the measurements under liquid conditions. The properties of the probe used in contact mode were as follows: resonance frequency: 13 kHz (±4 kHz); force constant: 0.2 N/m (±0.14 N/m); cantilever length: 450 *μ*m (±10 *μ*m); cantilever width: 38 *μ*m (±5 *μ*m); cantilever thickness: 2 *μ*m (±1 *μ*m); tip radius: 5 nm (±1 nm); and tip height: of 17 *μ*m (±2 *μ*m). The AFM probe tips were stabilized with DMEM or PBS for at least 10 minutes prior to scanning.

For AFM imaging, the cells were washed twice with filtered PBS and fixed for 20 min in 2.5% glutaraldehyde in PBS at room temperature and 5 ml PBS was added to culture dishes containing fixed cells. TECs fixed with glutaraldehyde were scanned in PBS solution at a resolution of 512 × 512 pixels, at a scan speed of 0.5 line/s. We fixed the cells with glutaraldehyde to get the high resolution image.

The cell stiffness was obtained from the force-distance (FD) curve on live TECs after 24 hours of Ang II or various ARBs application. The live cells were first identified using the contact imaging mode to determine the appropriate site for the FD curve without defects or impurities, and force data were obtained at locations with similar height to prevent edge effects. Live TECs were scanned in DMEM solution at a resolution of 256 × 256 pixels and a scan rate of 2 lines/s. The loading force was adjusted to below 1-2 nN to minimize cell damage. We calculated *K*_cell_, the cellular spring constant, by modeling the cell-tip interaction as two springs to quantify cell elasticity [16]. *K*_cell_ was defined as 1/*K*_cell_ = 1/*K*_eff_ − 1/*K*_cantilever_, where *K*_eff_ is the slope of the linear region of the FD curve for a cell and *K*_cantilever_ is determined from each cantilever using a clean culture dish containing DMEM. Data acquisition and image processing were performed with SPIP™ (Scanning Probe Image Processor Version 5.0.3, Image Metrology, Denmark). The fixation process could damage the cytoskeleton; we determine the FD curve in live cell without fixation. After FD curve measurements were completed, a second image was obtained to ensure that the cell had not shifted or was damaged.

### 2.3. Immunocytochemistry

TECs were washed in PBS before fixing in 4% paraformaldehyde for 1 hour at room temperature (RT). Cells were permeabilized with 0.1% Triton X-100 for 15 min at RT. Nonspecific antibody binding was blocked by incubating in 1% BSA for 30 min at RT, followed by overnight incubation with anti-rabbit phospho-myosin light chain (pMLC; Cell Signaling, #3671; 1 : 200 dilution) or anti-rabbit E-cadherin (Cell signaling, #3195; 1 : 200 dilution) antibodies at 4°C. The cells were then incubated with secondary antibodies consisting of anti-rabbit IgG FITC (Sigma, F0382; 1 : 500 dilution) for 2 hours at RT. Rhodamine phalloidin (Invitrogen, R415) is a high-affinity probe for F-actin that is synthesized from a mushroom toxin conjugated with the orange-fluorescent dye, tetramethylrhodamine (TRITC). F-actin staining was carried out for 2 hours at RT with rhodamine phalloidin (0.2 U/mL dilution). Finally, the slides were mounted using the VECTASHIELD HardSet Antifade Mounting Medium with DAPI (H-1500, Vector labs) and detected using a fluorescence or confocal microscope.

### 2.4. Statistical Analysis

The calculated spring constants of TECs are expressed as mean ± standard deviation (SD). ANOVA was used to evaluate the significance of the differences between the groups. All statistical analyses were carried out using SPSS software version 19.0 (SPSS Inc., Chicago, IL, USA); *p* < 0.05 was considered to be statistically significant.

## 3. Results

### 3.1. Morphological Changes in TECs after Treatment with Ang II and ARB Treatment

**Figure 1:**
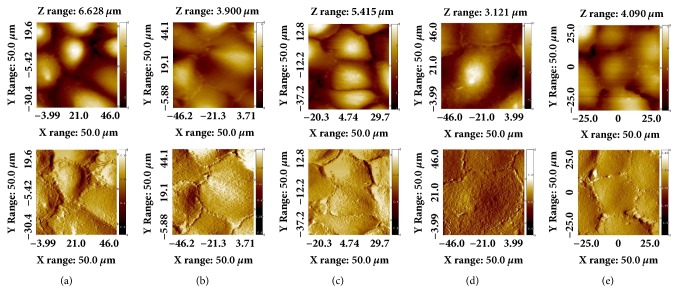
*Representative AFM topography (upper panels) and deflection images (lower panels) of fixed TECs.* (a) No treatment; (b) Ang II injection; (c) Ang II + Telmisartan; (d) Ang II + PD123319; (e) Ang II + Blebbistatin. AFM: atomic force microscopy; TECs: tubular epithelial cells; Ang II: angiotensin II.

Figure 1 shows representative AFM topography (upper panels) and deflection images (lower panels) taken from TECs fixed with glutaraldehyde in liquid conditions. After 24 hours in culture, control cells exhibited a typical epithelial cuboidal shape with cobblestone-like appearance. Cell bodies were convex, and many microvilli were regularly spread over the cell surface (Figure 1(a)). However, TECs cultured in Ang II revealed profound morphological changes. As shown in Figure 1(b), the cells became flattened and elongated and changed to a spindle-like shape. There were small bumps around the nucleus on the cell surface, and microvilli presence decreased when compared to control cells. Simultaneous incubation with telmisartan or blebbistatin disrupted the Ang II-induced morphological changes in majority of the cells, while retaining a cobblestone-like appearance, with the absence of hypertrophy and elongated morphology. (Figures 1(c) and 1(e)). PD12319, which was used as a negative control, had no significant effect on the morphological change of the TECs treated with Ang II (Figure 1(d)).

### 3.2. Mechanical Changes in Live TECs Induced by Ang II and ARBs

**Figure 2:**
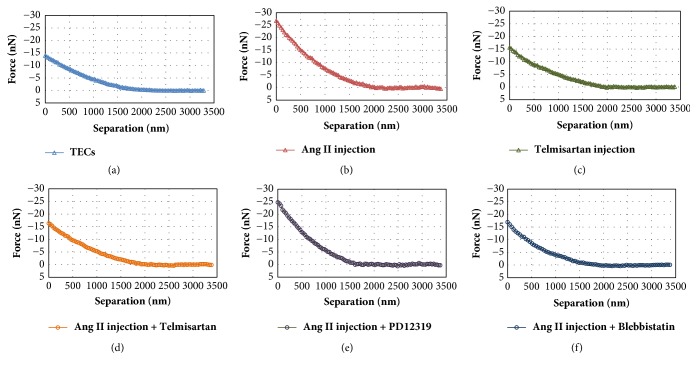
*Representative approach half of the force-distance curve in TECs.* (a) No treatment; (b) Ang II injection; (c) telmisartan injection; (d) Ang II + Telmisartan; (e) Ang II + PD123319; (f) Ang II + Blebbistatin. *K*_eff_ for calculated cellular spring constant (*K*_cell_) is obtained from the slope of the linear region of the each FD curve for a cell. TECs: tubular epithelial cells; Ang II: angiotensin II.

We calculated *K*_cell_, the cellular spring constant, from the FD curve in Figure 2. In this study, FD measurements were obtained for 30 cells in each group.  Table 1 shows the mean spring constants of TEC cell bodies. The spring constant of untreated TECs was 0.0093 ± 0.0025 N/m. However, the spring constant of Ang II-stimulated TECs increased to 0.0182 ± 0.0105 N/m (*p* < 0.0001 versus untreated cells). The spring constants significantly decreased with telmisartan or blebbistatin treatment (0.0096 ± 0.0030 N/m and 0.0118 ± 0.0023 Nm; *p* < 0.0001 and *p* < 0.0001 versus Ang II treated cells, respectively).

### 3.3. Immunofluorescent Findings

**Figure 3:**
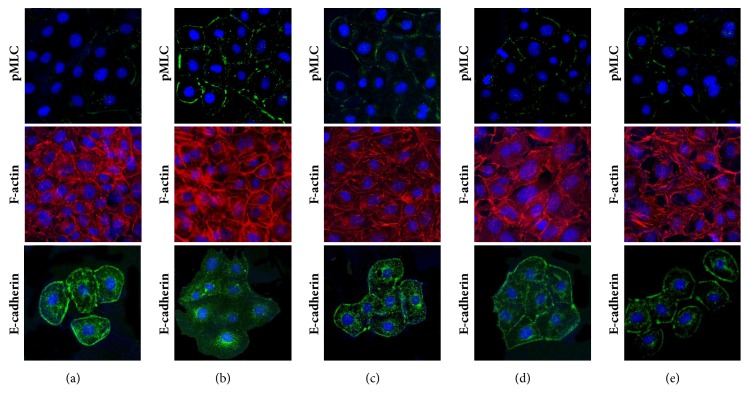
*Immunofluorescence staining for pMLC (upper panels), F-actin (mid panels), and E-cadherin (lower panels) of TECs.* (a) No treatment; (b) Ang II injection; (c) Ang II + Telmisartan; (d) Ang II + PD123319; (e) Ang II + Blebbistatin. pMLC: phospho-myosin light chain; TECs: tubular epithelial cells; Ang II: angiotensin II.

To confirm the transformation of TECs into a fibroblastic phenotype, the expression of pMLC, F-actin, and E-cadherin was investigated via immunofluorescent staining. As shown in Figure 3, pMLC (upper panels) and F-actin (mid panels) expressions markedly increased in TECs with Ang II treatment when compared to the control cells (Figures 3(a) and 3(b)). Conversely, E-cadherin expression (lower panels) markedly decreased in TECs with Ang II treatment when compared to the control cells (Figure 3(c)). As shown in Figures 3(d) and 3(e), telmisartan and blebbistatin blocked the Ang II-induced changes. Upon incubation with telmisartan or blebbistatin for 24 hours, pMLC and F-actin expression markedly decreased and those of E-cadherin markedly increased when compared to the expression of Ang II treated TECs.

## 4. Discussion

In the present study, we observed the Ang II-induced morphological and mechanical changes in TECs and investigated the effect of ARBs on Ang II-stimulated TECs. To our knowledge, this is the first study to visualize and characterize the changes in TECs induced by Ang II and ARBs using AFM. Our principle findings were as follows: (1) after treatment with Ang II, TECs exhibited notable morphological and mechanical changes; (2) Ang II caused the expression of EMT markers, including decreased expression of E-cadherin and increased expression of pMLC and F-actin; and (3) these changes and phenotypic conversion were disrupted by the addition of telmisartan.

Ang II has been reported to promote renal fibrosis by regulating ECM accumulation, inflammation, and cellular proliferation [9, 13]. Activation of the RAAS is also widely known to play a crucial role in the EMT of TECs [22]. Many studies have demonstrated that the suppression of RAAS results in renal protective effects and prevents renal fibrosis [10]. Therefore, understanding the changes in TECs and RAAS activation during the renal fibrosis process is important in understanding the mechanisms underlying renal damage.

Although several studies investigated changes in TECs after Ang II treatment, studies that demonstrate morphological and mechanical aspects are limited. In this study, we effectively examined the cell response to Ang II and ARBs with AFM imaging and FD curve measurement. As shown in Figure 1, we performed AFM imaging to directly observe the morphological changes in TECs after treatment with Ang II with or without ARBs. Although the fixation process could lead to cell damage, we used TECs fixed with glutaraldehyde to get high resolution images. After treatment with Ang II, TECs exhibited marked hypertrophy, lost their cobblestone-like morphology, and became elongated in shape, which is typical of fibroblasts. These morphological changes were accompanied with phenotypic changes. Immunofluorescent staining showed that TECs treated with Ang II lost their epithelial marker and newly acquired mesenchymal markers (Figure 3).

The structural and physical changes of TECs are difficult to visualize. Rabinovich et al. [23] found the existence of repulsive forces between the AFM tip and renal tubular epithelial cells. They reported that the oxalate treatment of renal TECs gave rise to increase elastic modulus of the cells. In the present study, we also used AFM to monitor and obtain mechanical properties and cell stiffness.  Table 1 shows the mechanical changes in TECs during Ang II-induced EMT. By using the AFM spring constant, we showed that the contractile response of TECs can generate stiffness, which may deform the surrounding ECM or exchange in tissue containing a TEC layer. In addition to the methods used in this study to calculate cells' stiffness, more advanced methods have been suggested [24, 25]. To evaluate cell's elasticity, they determined Young's modulus by AFM. In this study, we calculated the spring constant to quantify cell elasticity. In our future study, we will also consider apply these methods.

As mentioned above, we revealed that Ang II-induced morphological and mechanical changes that were attenuated via telmisartan treatment. It is now widely recognized that RAAS blockade by ARB exhibits a therapeutic effect in renal injury [10]. Our results suggest that telmisartan may disrupt Ang II-induced renal damage by reducing the morphological changes and contraction of TECs. Several studies reported that ARBs diminished renal fibrosis and the expression of profibrotic growth factors such as transforming growth factor-*β* and connective tissue growth factor [7, 9, 13]. The reduction in molecular and mechanical changes of TECs in our study were presumed to be due to telmisartan-induced biochemical modification. The morphological and mechanical changes of cells have been reported to be associated with the changes of cytoskeletal structures. Ang II could affect the increase of cytoskeleton activity and lead to the changes of the elastic modulus of the cell [26]. These dynamics of cytoskeletal structure could be considered the cause of cell stiffness, but further studies are needed to confirm their contribution.

## 5. Conclusion

In summary, we observed morphological changes in TECs induced by Ang II treatment with the help of AFM imaging. Furthermore, the mechanical changes in TECs were evaluated using FD curve analysis. We also demonstrated that these morphological and mechanical changes were effectively prevented by telmisartan treatment. Although the mechanisms underlying these physical changes in TECs have not yet been fully elucidated, AFM could provide noninvasive measurements of the cellular processes in TECs.

## Figures and Tables

**Table 1:** Calculated spring constant of live TECs (*n* = 30) before and 20 min after Ang II stimulation with or without telmisartan, PD123319, and blebbistatin.

	*K*cell (N/m,)	Statistical significance	
		vs. No treatment	vs. Ang II
No treatment	0.0093 ± 0.0025		*p* < 0.0001
Ang II injection	0.0182 ± 0.0105	*p* < 0.0001	
Telmisartan injection	0.0104 ± 0.0021		*p* < 0.0001
Ang II injection + Telmisartan	0.0096 ± 0.0030		*p* < 0.0001
Ang II injection + PD12319	0.0220 ± 0.0086	*p* < 0.005	
Ang II injection + Blebbistatin	0.0118 ± 0.0023	*p* < 0.01	*p* < 0.0001

TECs, tubular epithelial cells; Ang II, angiotensin II.

## Data Availability

The data used to support the findings of this study are available from the corresponding author upon request.

## References

[1] Boor P., Ostendorf T., Floege J. (2010). Renal fibrosis: novel insights into mechanisms and therapeutic targets. *Nature Reviews Nephrology*.

[2] Zeisberg M., Neilson E. G. (2010). Mechanisms of tubulointerstitial fibrosis. *Journal of the American Society of Nephrology*.

[3] Cantaluppi V., Quercia A. D., Dellepiane S., Ferrario S., Camussi G., Biancone L. (2014). Interaction between systemic inflammation and renal tubular epithelial cells. *Nephrology Dialysis Transplantation*.

[4] Lee S. B., Kalluri R. (2010). Mechanistic connection between inflammation and fibrosis. *Kidney International Supplements*.

[5] Neilson E. G. (2006). Mechanisms of disease: fibroblasts—a new look at an old problem. *Nature Clinical Practice Nephrology*.

[6] Mao H., Li Z., Zhou Y. (2008). HSP72 attenuates renal tubular cell apoptosis and interstitial fibrosis in obstructive nephropathy. *American Journal of Physiology-Renal Physiology*.

[7] Ruiz-Ortega M., Rupérez M., Esteban V. (2006). Angiotensin II: a key factor in the inflammatory and fibrotic response in kidney diseases. *Nephrology Dialysis Transplantation*.

[8] Remuzzi G., Perico N., Macia M., Ruggenenti P. (2005). The role of renin-angiotensin-aldosterone system in the progression of chronic kidney disease. *Kidney International*.

[9] Mezzano S. A., Ruiz-Ortega M., Egido J. (2001). Angiotensin II and renal fibrosis. *Hypertension*.

[10] Murphy A. M., Wong A. L., Bezuhly M. (2015). Modulation of angiotensin II signaling in the prevention of fibrosis. *Fibrogenesis & Tissue Repair*.

[11] Siragy H. M. (2004). AT_1_ and AT_2_ receptor in the kidney: role in health and disease. *Seminars in Nephrology*.

[12] Chen J., Chen J.-K., Harris R. C. (2012). Angiotensin II induces epithelial-to-mesenchymal transition in renal epithelial cells through reactive oxygen species/Src/caveolin-mediated activation of an epidermal growth factor receptor-extracellular signal-regulated kinase signaling pathway. *Molecular and Cellular Biology*.

[13] Carvajal G., Rodríguez-Vita J., Rodrigues-Díez R. (2008). Angiotensin II activates the Smad pathway during epithelial mesenchymal transdifferentiation. *Kidney International*.

[14] Binnig G., Quate C. F., Gerber C. (1986). Atomic force microscope. *Physical Review Letters*.

[15] Gad M., Ikai A. (1995). Method for immobilizing microbial cells on gel surface for dynamic AFM studies. *Biophysical Journal*.

[16] Volle C. B., Ferguson M. A., Aidala K. E., Spain E. M., Núñez M. E. (2008). Quantitative changes in the elasticity and adhesive properties of Escherichia coli ZK1056 prey cells during predation by Bdello vibrio bacteriovorus 109J. *Langmuir*.

[17] Oberleithner H., Schneider S., Länner J., Henderson R. M. (1996). Viewing the renal epithelium with the atomic force microscope. *Kidney and Blood Pressure Research*.

[18] Jeong K. H., Lee S. H. (2012). A new technical approach to monitor the cellular physiology by atomic force microscopy. *Electrolytes & Blood Pressure*.

[19] Lee G.-J., Park E.-J., Choi S. (2010). Observation of angiotensin II-induced changes in fixed and live mesangial cells by atomic force microscopy. *Micron*.

[20] Jeong K.-H., Lee T.-W., Ihm C.-G. (2013). Real-time monitoring of the effects of telmisartan on angiotensin II-induced mechanical changes in live mesangial cells using atomic force microscopy. *Kidney and Blood Pressure Research*.

[21] Lee G.-J., Kim J.-H., Kang S.-W., Chae S.-J., Jeong K.-H., Park H.-K. Ultrastructure and mechanical property of angiotensin II-stimulated tubular cells pretreated with angiotensin II type 1 receptor blocker.

[22] Liu Y. (2004). Epithelial to mesenchymal transition in renal fibrogenesis: pathologic significance, molecular mechanism, and therapeutic intervention. *Journal of the American Society of Nephrology*.

[23] Rabinovich Y., Esayanur M., Daosukho S., Byer K., El-Shall H., Khan S. (2005). Atomic force microscopy measurement of the elastic properties of the kidney epithelial cells. *Journal of Colloid and Interface Science*.

[24] Lekka M., Laidler P., Gil D., Lekki J., Stachura Z., Hrynkiewicz A. Z. (1999). Elasticity of normal and cancerous human bladder cells studied by scanning force microscopy. *European Biophysics Journal*.

[25] Sharma S., Gimzewski J. K. (2016). Application of AFM to the nanomechanics of cancer. *MRS Advances*.

[26] Cuerrier C. M., Benoit M., Guillemette G., Gobeil F., Grandbois M. (2009). Real-time monitoring of angiotensin II-induced contractile response and cytoskeleton remodeling in individual cells by atomic force microscopy. *Pflügers Archiv—European Journal of Physiology*.

